# Tanshinone IIA attenuates atherosclerosis via inhibiting NLRP3 inflammasome activation

**DOI:** 10.18632/aging.202202

**Published:** 2020-11-16

**Authors:** Jiexia Wen, Yumei Chang, Shanshan Huo, Wenyan Li, Heling Huang, Yunhuan Gao, Hongyu Lin, Jianlou Zhang, Yonghong Zhang, Yuzhu Zuo, Xuebin Cao, Fei Zhong

**Affiliations:** 1 College of Veterinary Medicine and College of Animal Science and Technology, Hebei Veterinary Biotechnology Innovation Center, Hebei Agricultural University, Baoding 071001, Hebei, China; 2 Department of Cardiology, 252 Hospital of Chinese PLA, Baoding 071000, Hebei, China; 3 Department of Central Laboratory, First Hospital of Qinhuangdao, Qinhuangdao 066000, Hebei, China; 4 Department of Biology, College of Basic Medicine, Hebei University, Baoding 071000, Hebei, China

**Keywords:** atherosclerosis, inflammasome, oxidized LDL, tanshinone IIA, LOX-1

## Abstract

Tanshinone IIA (Tan IIA) possesses potent anti-atherogenic function, however, the underlying pharmacological mechanism remains incompletely understood. Previous studies suggest that oxidized LDL (oxLDL)-induced NLRP3 (NOD-like receptor (NLR) family, pyrin domain-containing protein 3) inflammasome activation in macrophages plays a vital role in atherogenesis. Whether the anti-atherogenic effect of Tan IIA relies on the inhibition of the NLRP3 inflammasome has not been investigated before. In this study, we found that Tan IIA treatment of high-fat diet fed ApoE-/- mice significantly attenuated NLRP3 inflammasome activation in vivo. Consistently, Tan IIA also potently inhibited oxLDL-induced NLRP3 inflammasome activation in mouse macrophages. Mechanically, Tan IIA inhibited NF-κB activation to downregulate pro-interleukin (IL) -1β and NLRP3 expression, and decreased oxLDL-induced expression of lectin-like oxidized LDL receptor-1 (LOX-1) and cluster of differentiation 36 (CD36), thereby attenuating oxLDL cellular uptake and subsequent induction of mitochondrial and lysosomal damage — events that promote the NLRP3 inflammasome assembly. Through regulating both the inflammasome ‘priming’ and ‘activation’ steps, Tan IIA potently inhibited oxLDL-induced NLRP3 inflammasome activation, thereby ameliorating atherogenesis.

## INTRODUCTION

Tanshinone IIA (Tan IIA) is one of the major therapeutic components of *Salvia miltiorrhiza Bunge*. It is widely used to treat cardiovascular diseases, such as angina pectoris, myocardial infarction and other heart disorders due to its anti-atherosclerotic (AS) effects [1, 2]. However, the mechanism of anti-atherogenesis of Tan IIA remains incompletely understood. This is not only because the pharmacological functions of Tan IIA are not entirely clear [3], but the mechanisms of AS formation are also not fully elucidated [4]. In recent decades, the dysregulated sterile inflammation was identified to be related to the development of many inflammatory, metabolic and degenerative diseases [5]. The inflammasome-mediated inflammation plays a crucial role in the pathology of some cardiovascular diseases [6–8]. AS, a major cardiovascular disease, is a multifactorial disorder in which sterile inflammation was shown to play a pathogenic role [9]. It has been shown that aberrant macrophage activation and subsequent uncontrolled production of pro-inflammatory cytokines in AS plaque mediate AS progression [10]. Recent studies revealed that the NLRP3 inflammasome activation by oxidized low density lipoprotein (oxLDL) and its derived cholesterol crystal (ChC) is a key pathogenic event that drives initiation of sterile inflammation in AS [10–17].

The inflammasomes are a group of intracellular multimeric protein complexes that are mainly responsible for processing and maturation of the inflammatory cytokines IL-1β and IL-18, and thereby constitutes the first line of the host defense [18, 19]. A typical inflammasome usually consists of an inflammasome sensor protein, the adaptor protein apoptosis-associated speck-like protein containing CARD (ASC) and pro-caspase-1. Several inflammasome sensor proteins have been identified to date, including Nod-like receptors (NLRs), RIG-I-like receptors (RLRs) and AIM2-like receptors (ALRs) [20]. NLR Family Pyrin Domain Containing 3 (NLRP3) is the most studied and best characterized NLRs in monocytes/macrophages [21], which senses many exogenous pathogenic stimuli [22–24] as well as endogenous ‘‘danger’’ signals [15, 25–28]. Since these inflammatory stimuli are functionally and structurally unrelated, it is believed that they in turn activate common downstream signaling intermediates (e.g. mitochondrial damage, lysome rupture and ion mobilization) that eventually result in the inflammasome assembly and activation. The inflammasome activation leads to caspase 1 autocleavage and activation, which then cleaves pro-IL-1β and pro-IL-18 into their bioactive forms respectively, thereby initiating inflammation [19].

During atherosclerosis, oxLDL was taken up by macrophages via scavenger receptors, including lectin-like oxidized LDL receptor-1 (LOX-1), scavenger receptor A1 (SR-A1) and cluster of differentiation 36 (CD36) [29, 30]. LOX-1 is highly expressed on vascular endothelial cells, smooth muscle cells and macrophages [31–33]. Upon binding by oxLDL, LOX-1-mediated uptake of oxLDL induces mitochondrial damage causing mitochondrial reactive oxygen species (mtROS) accumulation, and mitochondrial DNA (mtDNA) damage and release, both mtROS and mtDNA can engages NLRP3 [34–36]. Another important oxLDL receptor is CD36 that mediates soluble oxLDL-Ch nucleation into particulate ChC (Cholesterol crystal) in endo-lysosome. The “undigested” particulate ChC breaches the membrane of late endosomes/lysosomes, resulting in cathepsin B release from lysosome into cytosol [37]—an event shown to induce NLRP3 inflammasome assembly and robust IL-1β production, thereby promoting AS development [8].

Although Tan IIA was shown to antagonize LPS-induced NF-κB activation [38], ROS accumulation [39] and NLRP3 activation in cardiac myocytes [7] and scavenger receptor expression in macrophages [40], how it inhibits AS has remained incompletely understood. Here we show that Tan IIA potently inhibited oxLDL-induced NLRP3 inflammasome activation by attenuating both the priming and activation signals. Mechanically, Tan IIA inhibited NF-κB activation to downregulate pro-IL-1β and NLRP3 expression, and decreased oxLDL-induced expression of LOX-1 and CD36, attenuating oxLDL cellular uptake and subsequent induction of mitochondrial damage and mtROS accumulation. In summary, our results have revealed previously unrecognized mechanisms underlying Tan IIA’s potent anti-AS effects, and thereby provide a molecular basis for its clinical use to treat AS.

## RESULTS

### Tan IIA inhibits atherosclerosis and IL-1β/IL-18 production in vivo

To confirm the anti-AS effect of Tan IIA *in vivo*, we tested whether the administration of Tan IIA (Figure 1A) could inhibit atherogenesis in the *ApoE*^-/-^ mice fed with a high-cholesterol diet (HCD). Histomorphological analyses revealed that the AS plaque size in the aorta of the Tan IIA-treated mice (~ 20 % of total area) were significantly lower than that of untreated mice (~ 45 % of total area) (Figure 1B, 1C). Consistently, Tan IIA treatment also strongly reduced cross-section areas of the plaque in HCh-diet-fed mice relative to untreated controls (Figure 1D, 1E). High levels of total Ch (T-Ch), low-density-lipoprotein Ch (LDL-Ch) and triglyceride (TG) in the sera are widely considered as the major risk factors of AS. Although Tan IIA had little effect on serum T-Ch and HDL-Ch levels (Figure 1F, 1G), it significantly decreased the amounts of LDL-Ch and TG in the sera of HCD-fed ApoE-deficient mice (Figure 1H, 1I), with the strongly reduction seen in LDL-Ch (~40%). These results have therefore collectively confirmed Tan IIA’s potent anti-AS activity *in vivo*.

**Figure 1:**
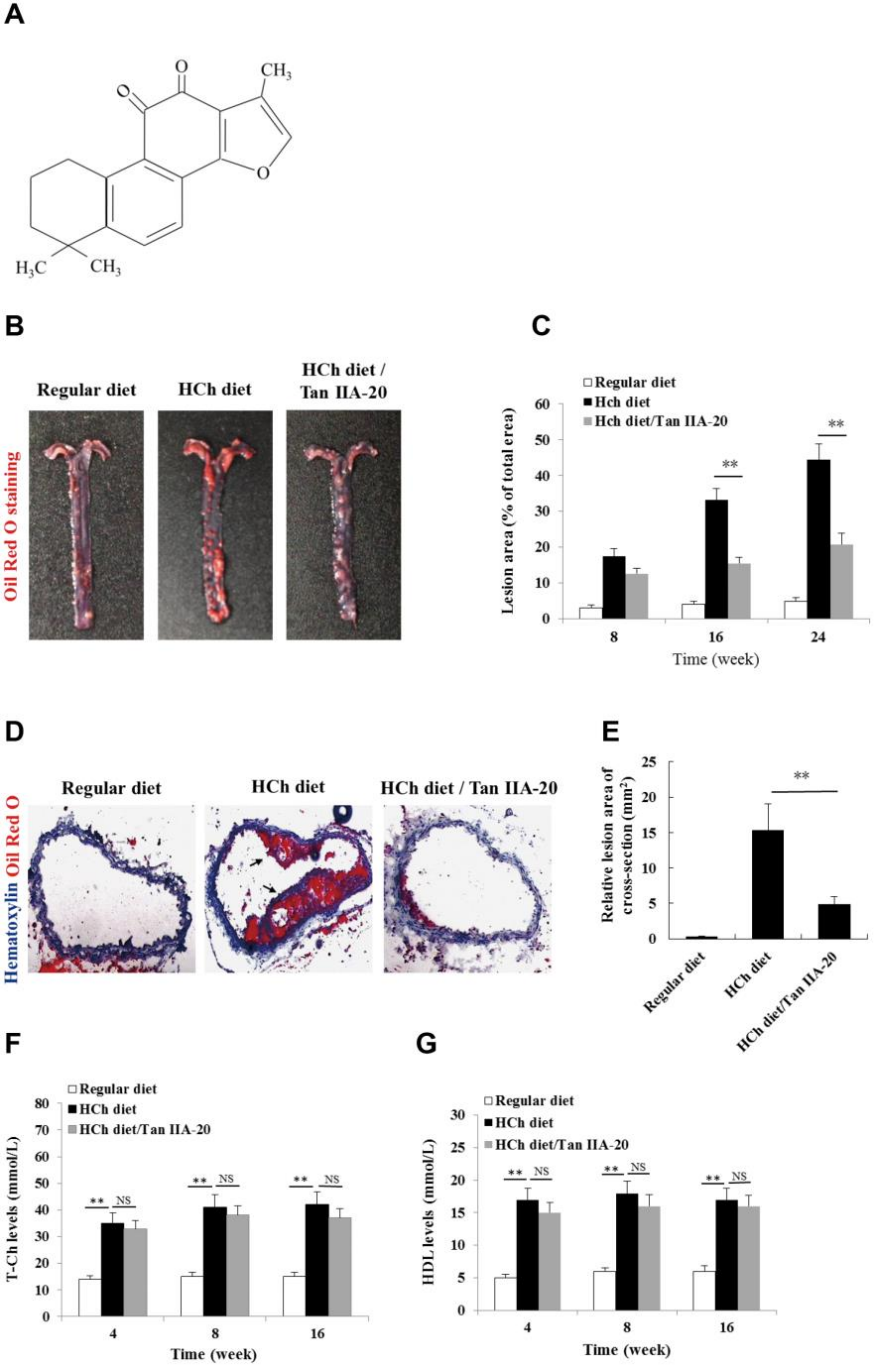
**Effects of Tan IIA on HCh diet-induced AS lesion formation, serum lipid levels and cytokine productions in ApoE-/- mice.** (**A**) Molecular structure of Tanshinone IIA. (**B**) AS lesions stained by Oil Red O in the aorta of ApoE-deficient mice fed on the different diets (regular diet, HCh diet and HCh diet plus Tan IIA (20 mg/kg/day)). (**C**) Percentage of the lesion area of the aorta was calculated as the ratio of the lesion area to the total area. (**D**) Histological images of frozen section of the lesions stained with Oil Red O and Haematoxylin/eosin (HE). (**E**) Relative lesion area of the section of AS in the aorta. Average areas of AS lesions were calculated from 12 sections in ApoE-deficient mice fed on the different diets. (**F**–**G**) Levels of T-Ch and HDL-Ch in the sera of ApoE-deficient mice fed with different diets for 16 weeks.

**Figure 1:**
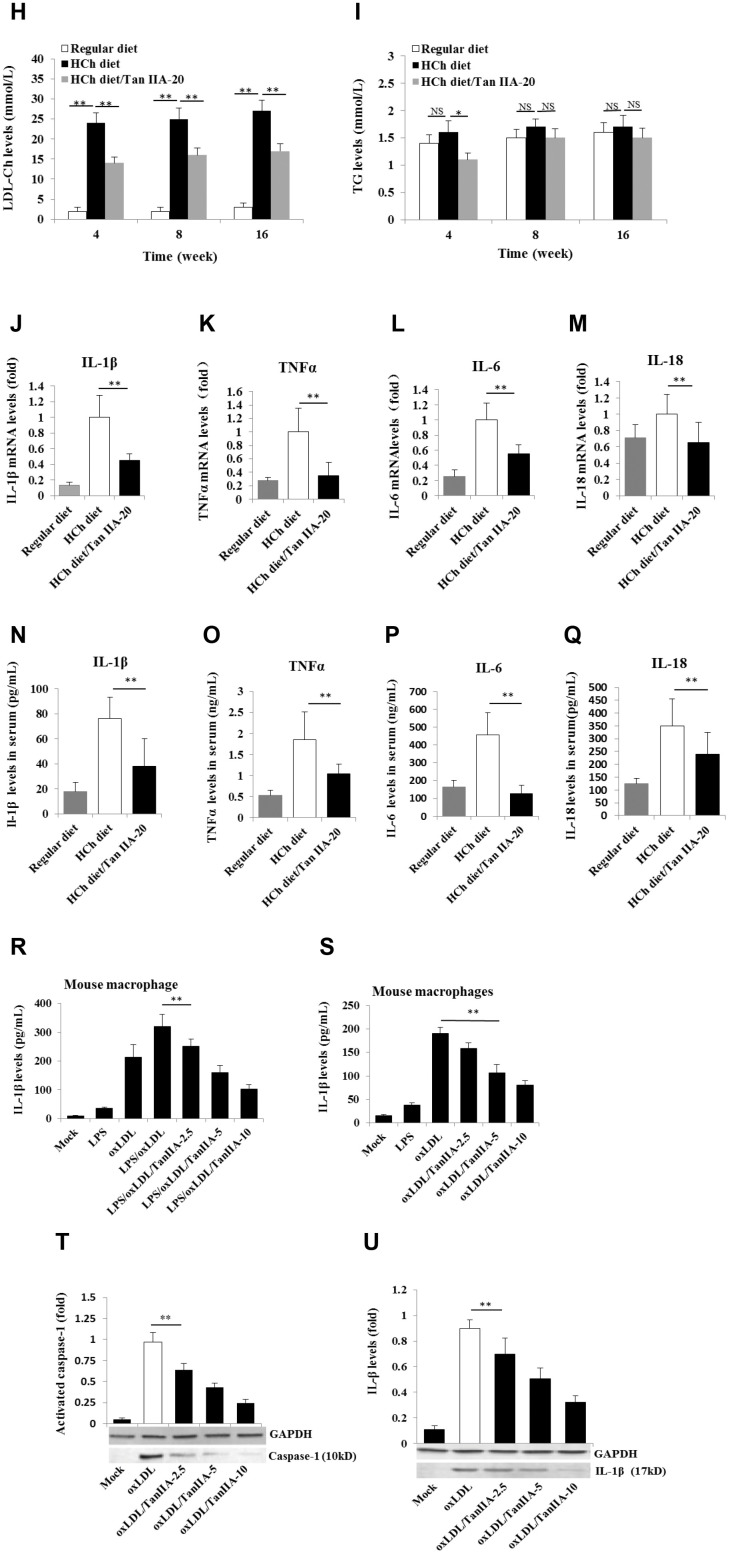
**Effects of Tan IIA on HCh diet-induced AS lesion formation, serum lipid levels and cytokine productions in ApoE-/- mice.** (**H**–**I**) Levels of LDL-Ch and TG in the sera of ApoE-deficient mice fed with different diets for 16 weeks. Oxidized LDL-induced and Tan IIA inhibited cytokine (IL-1β, TNFα, IL-6 and IL-18) productions at mRNA levels measured by qRT-PCR in the aortic tissues (**J**–**M**) and at protein levels determined by ELISA in the sera (**N**–**Q**). (**R**, **S**) Effects of Tan IIA (2.5, 5, 10 μg/mL) on oxLDL-induced IL-1β productions in the culture media of LPS-primed and un-primed mouse B6 macrophages, respectively. (**T**, **U**) Effects of Tan IIA on oxLDL-induced caspase-1 activation (10 kDa) and IL-1β maturation (17 kDa) in mouse B6 macrophages.

OxLDL- and ChC-induced inflammasome activation was recently shown to be a key pathogenic event that promotes AS lesion formation and progression [15, 16]. Consistent with this notion, mice deficient in inflammasome signaling are resistant to atherogenesis [15]. To investigate whether Tan IIA inhibits inflammasome activation *in vivo*, we analyzed the the production of inflammasome-dependent cytokines, IL-1β and IL-18, in the aorta of the HCD-fed ApoE-deficient mice with or without Tan IIA treatment. Indeed, Tan IIA significantly inhibited HCD-induced IL-1β and IL-18 production both in the aortic tissues (Figure 1J, 1M) and in the sera (Figure 1N, 1Q) of ApoE-deficient mice, suggesting that Tan IIA can inhibit inflammasome activation. Interestingly, we also noted reduced levels of TNFα and IL-6 after Tan IIA treatment (Figure 1K, 1L, 1O, 1P), which indicates that Tan IIA is likely to interfere with NF-κB signaling in general.

### Tan IIA inhibits oxLDL-induced NLRP3 inflammasome activation

To confirm our above *in vivo* results, we further tested whether Tan IIA could inhibit IL-1β production in mouse macrophages *in vitro*. We first noted that oxLDL itself can act both as priming and activation signals to induce mature IL-1β production (Figure 1R, 1S) from mouse bone marrow-derived macrophages (BMDMs). We next reconfirmed that oxLDL activates NLRP3 inflammasome, not other inflammasomes, because BMDMs deficient in NLRP3, ASC or Casp1, but not AIM2 nor NLRC4, failed to induce IL-1β release upon oxLDL stimulation (Supplementary Figure 1A–1F). Importantly, pretreatment of Tan IIA significantly reduced oxLDL-induced IL-1β release (Figure 1U). Consistently, caspase-1 activation, as shown by the presence of autocleaved p10 fragments, was also reduced upon Tan IIA treatment (Figure 1T). These data collectively suggest that Tan IIA inhibits oxLDL-induced inflammasome activation in cultured macrophages.

### Tan IIA inhibits the inflammasome priming signaling pathway

**Figure 2:**
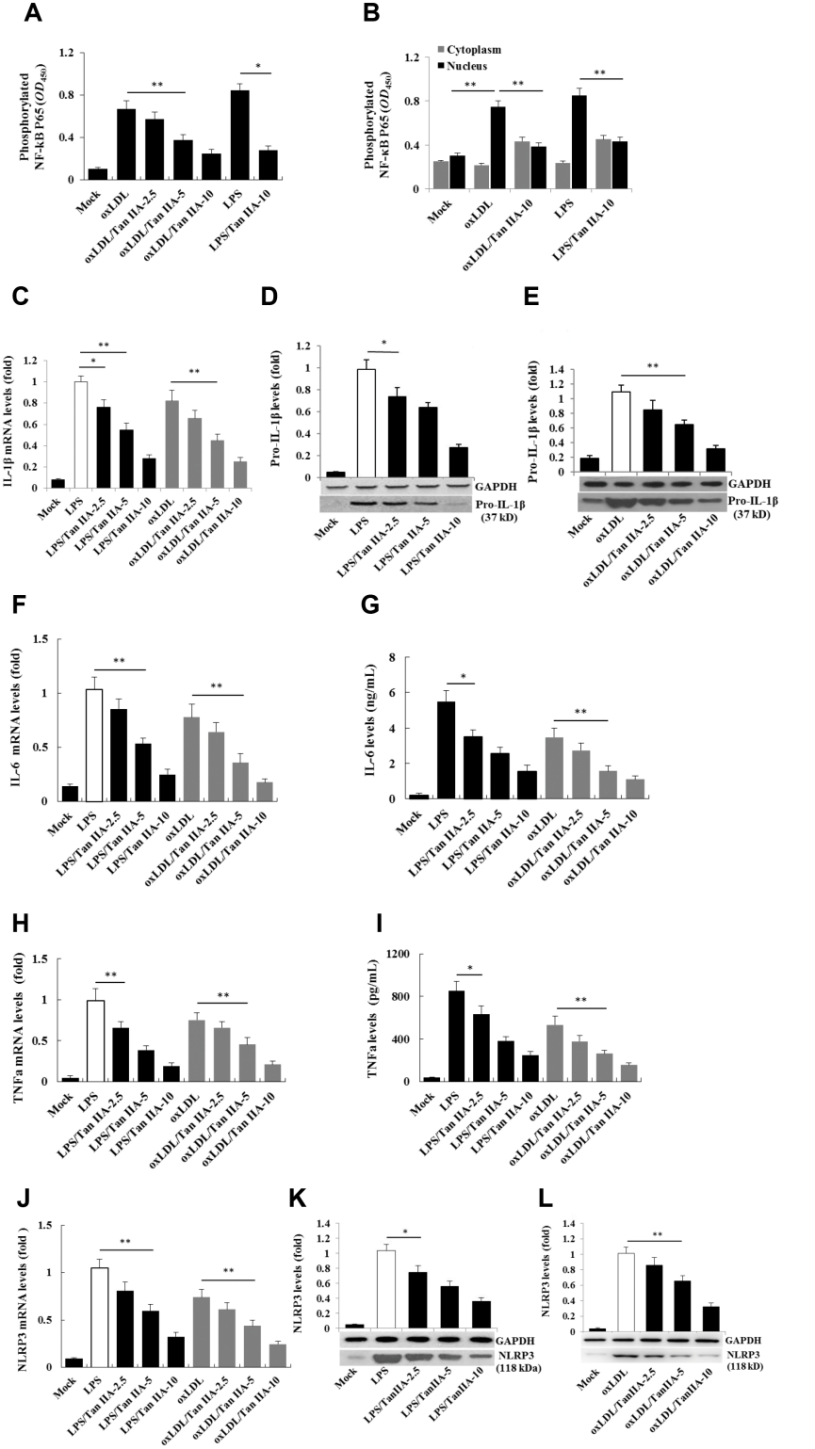
**Tan IIA inhibits LPS- and oxLDL-induced pro-inflammatory cytokine and NLRP3 production in macrophages.** (**A, B**) Effects of Tan IIA on LPS- or oxLDL-induced NF-κB P56 activity in the cytoplasm and nucleus of mouse B6 macrophages. (**C**) Effects of Tan IIA at the different doses (2.5, 5 and 10 μg/mL) on pro-IL-1β expressions by qRT-PCR detection in LPS- and oxLDL-induced mouse B6 macrophages. (**D**, **E**) Effects of Tan IIA on pro-IL-1β expressions detected by Western blot in LPS- and oxLDL-induced B6 macrophages, respectively. (**F**, **G**) Effects of Tan IIA on IL-6 expression in the cells by RT-PCR and in the culture medium by ELISA, respectively, in LPS- and oxLDL-induced B6 macrophages. (**H**, **I**) Effects of Tan IIA on TNFα expression in the cells by qRT-PCR and in the culture medium by ELISA, respectively, in LPS- and oxLDL-induced B6 macrophages. (**J**) Effects of Tan IIA on NLRP3 expressions by qRT-PCR detection in LPS- and oxLDL-induced mouse B6 macrophages. (**K**, **L**) Effects of Tan IIA on NLRP3 expressions in the culture medium by Western blot in LPS- and oxLDL-induced B6 macrophages, respectively.

Two signals are required for the production of biologically active IL-1β: the NF-κB-mediated priming signal for pro-IL-1β and NLRP3 expression, and Nod-like receptor-mediated activation signal for IL-1β maturation/release [19, 20, 18]. To test whether Tan IIA inhibits the priming step, the immortalized bone marrow-derived macrophages from C57BL/6 mice (hereafter referred as B6 macrophages) were pretreated with different doses of Tan IIA, followed by stimulation with LPS for 6 h or oxLDL for 24 h. We found that LPS and oxLDL induced NF-κB P65 phosphorylation in the cells and nucleus (Figure 2A, 2B) and subsequent upregulation of pro-IL-1β (Figure 2C–2E), IL-6 (Figure 2F, 2G), TNFα (Figure 2H, 2I) and NLRP3 (Figure 2J–2L) expression. These results suggest that oxLDL can activate both signals for inflammasome activation, while the Tan IIA strongly inhibited the priming signal by preventing NF-κB activation.

### oxLDL activates the NLRP3 inflammasome via LOX-1 and CD36

**Figure 3:**
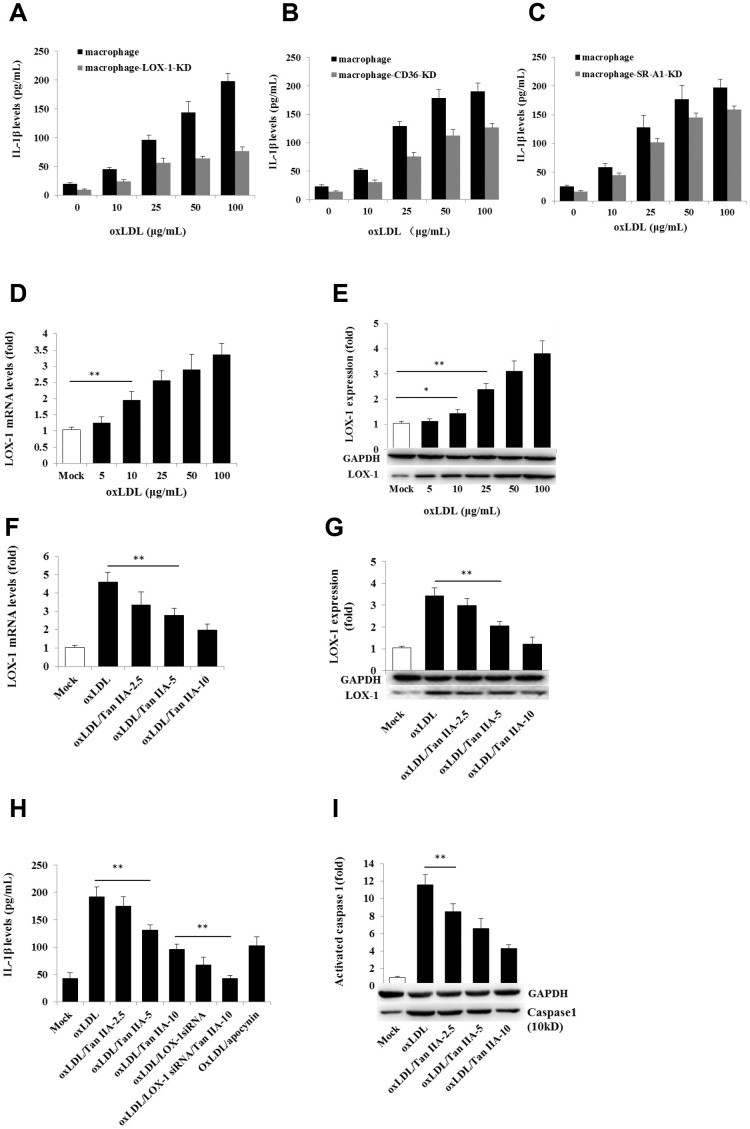
**Effects of the LOX-1, CD36 and SR-A1 siRNAs on oxLDL-induced IL-1β productions and effects of oxLDL and Tan IIA on LOX-1 expression and LOX-1-mediated caspase-1activation/IL-1β release.** (**A**–**C**) Effects of lentivirus-mediated LOX-1, CD36 and SR-A1 siRNAs (MOI = 30) on oxLDL-induced IL-1β productions in mouse macrophages measured by ELISA. (**D**, **E**) Effects of oxLDL on LOX-1 expression in macrophages measured by qRT-PCR and Western blot, respectively. (**F**, **G**) Effects of Tan IIA on oxLDL-induced LOX-1 expression determined by qRT-PCR and Western blot, respectively. (**H**) Effects of Tan IIA on oxLDL-induced LOX-1-mediated IL-1β expression in macrophages. (**I**) Effects of Tan IIA on oxLDL-induced LOX-1-mediated caspase-1 activation in macrophages.

We next further investigated the mechanism by which oxLDL activates the NLRP3 inflammasome and how Tan IIA inhibited such a process. Cellular uptake of oxLDL is required for activating macrophages. Three membrane-bound scavenger receptors (LOX-1, CD36 and SR-A1) were implicated in oxLDL cellular uptake and foam cell formation [19, 20, 18]. To determine which of these three receptors is dominantly involved in oxLDL-induced NLRP3 activation, lentivirus-mediated small interfering RNA (siRNA) against mouse LOX-1, CD36 and SR-A1 (Supplementary Table 2) were used to knock down (KD) their expressions in the macrophages, respectively (Supplementary Figure 2A–2C). We found that LOX-1 and CD36, but not SR-A1 siRNA potently inhibited IL-1β productions (Figure 3A–3C), suggesting that oxLDL-induced NLRP3 activation mainly requires LOX-1 and CD36 mediated cellular uptake pathways.

### Tan IIA inhibits oxLDL-induced LOX-1 expression and subsequent NLRP3 activation

Next we investigated whether Tan IIA regulates LOX-1. To this end, we pretreated WT and LOX-1-KD macrophages with Tan IIA and then stimulated them with oxLDL. As shown in Figure 3D and 3E, oxLDL potently induced LOX-1 expression in a dose-dependent manner—an effect that was inhibited by Tan IIA pretreatment (Figure 3F, 3G). Importantly, we found that Tan IIA also inhibited the release of IL-1β from macrophages (Figure 3H), and reduced caspase 1 activation (Figure 3I), indicating that Tan IIA can inhibit oxLDL-induced LOX-1-mediated inflammasome activation.

### Tan IIA prevents oxLDL-induced mitochondrial damage

LOX-1-mediated cellular uptake pathway was previously shown to trigger mitochondria damage and subsequent production of ROS [37], which were shown to be an important activator of NLRP3 inflammasome [36, 42]. We then speculated that Tan IIA may inhibit oxLDL-induced LOX-1-mediated ROS generation, thereby keeping the NLRP3 inflammasome activation in check. To test this, WT and LOX-1-KD macrophages were treated with Tan IIA (10 μg/mL) followed by stimulation with oxLDL (50 μg/mL). The amounts of ROS were then quantified using the 2´, 7´-Dichlorofluorescein diacetate (DCFH-DA) probe. The results showed that oxLDL treatment induced robust ROS generation in WT macrophage relative to untreated cells (Figure 4A1, A2), and that deficiency in LOX-1 drastically attenuated ROS production (Figure 4A3). Of note, prior treatment of Tan IIA significantly inhibited ROS production induced by oxLDL or ATP—a classical NLRP3 activator (Figure 4A4–A6).

**Figure 4:**
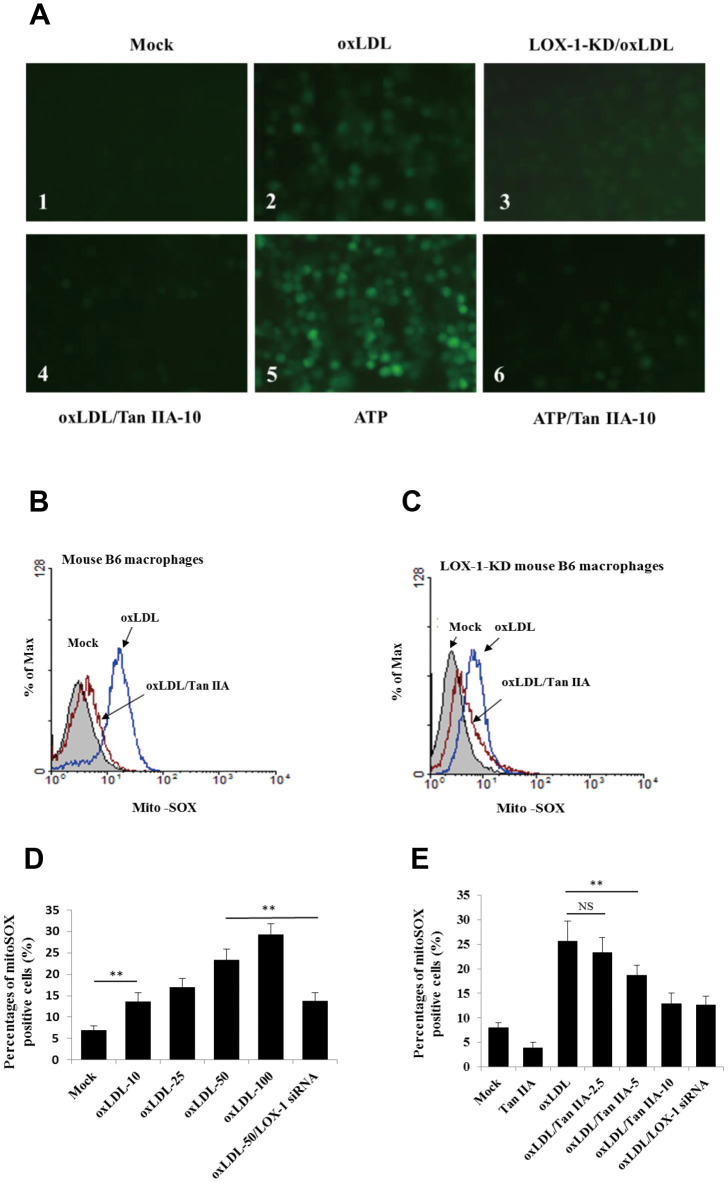
**Tan IIA inhibits oxLDL-induced LOX-1-mediated ROS production and mitochondrial damage in macrophages.** (**A**) Oxidized LDL induction and Tan IIA inhibition on ROS generation detected using ROS fluorescence detector DCFH-DA (ATP treatment as a positive control). (**B**, **C**) Oxidized LDL induction and Tan IIA inhibition on mito-ROS production detected with MitoSOX (Invitrogen) using a flow cytometer in mouse B6 macrophages and LOX-1-KD B6 macrophages, respectively. (**D**) Mito-ROS production in the B6 macrophages induced by oxLDL at the different doses (0, 10, 25, 50, 100 μg/mL).

**Figure 4:**
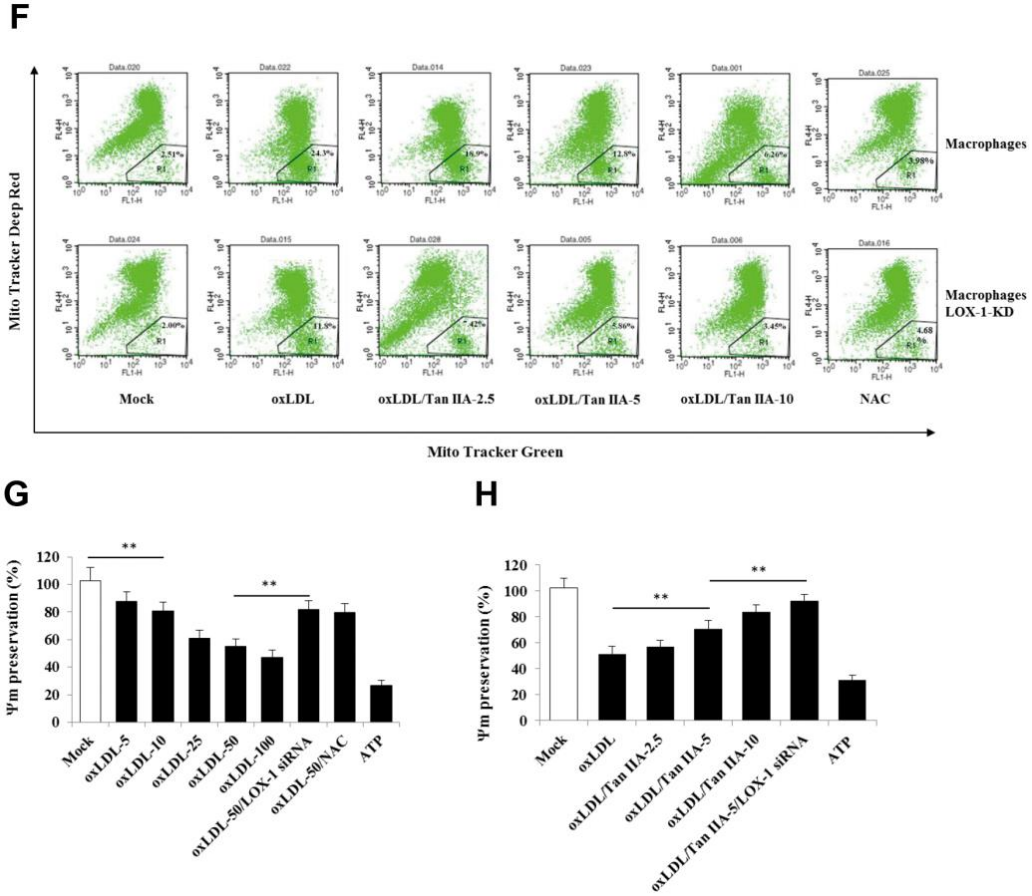
**Tan IIA inhibits oxLDL-induced LOX-1-mediated ROS production and mitochondrial damage in macrophages.** (**E**) Inhibition of Tan IIA on oxLDL-induced mito-ROS production in the B6 macrophages. (**F**) The oxLDL-induced mitochondria damage and Tan IIA inhibition on it in mouse B6 macrophages and LOX-1-KD macrophages. MitoTracker Deep Red for health mitochondria staining and MitoTracker Green FM for total mitochondria staining. NAC is a ROS inhibitor. (**G**) The oxLDL-induced mitochondrial membrane potential loss. (**H**) Tan IIA inhibition on oxLDL-induced mitochondrial membrane potential loss. ATP as a positive control for oxLDL-induced the loss of mitochondrial membrane potential.

It has been demonstrated that mitochondrial damage and subsequent mitochondrial ROS (mtROS) production are key events during NLRP3 inflammasome activation [36, 42]. Indeed, we found that oxLDL induced robust mtROS production—as quantified by a mitochondrial superoxide probe MitoSOX—in WT macrophages, while to a less extent, in LOX-1-deficient macrophages (Figure 4B–4F). Consistently, LOX-1 deficient macrophages had less mitochondrial damage and loss of mitochondrial membrane potential (ΔΨm) relative to WT cells after oxLDL stimulation (Figure 4G, 4H). This suggests that LOX-1 at least partially contributes to oxLDL-induced mitochondrial damage and subsequent mtROS production. Notably, Tan IIA pretreatment effectively inhibited the oxLDL-induced mitochondrial damage and mtROS generation both in WT and LOX-1-deficient macrophages (Figure 4B–4F). These results suggest that Tan IIA inhibits oxLDL-induced LOX-1 mediated mitochondrial damage, thereby preventing the activation of the NLRP3 inflammasome.

### The inhibitory effect of Tan IIA on NLRP3 inflammasome also involves CD36

**Figure 5:**
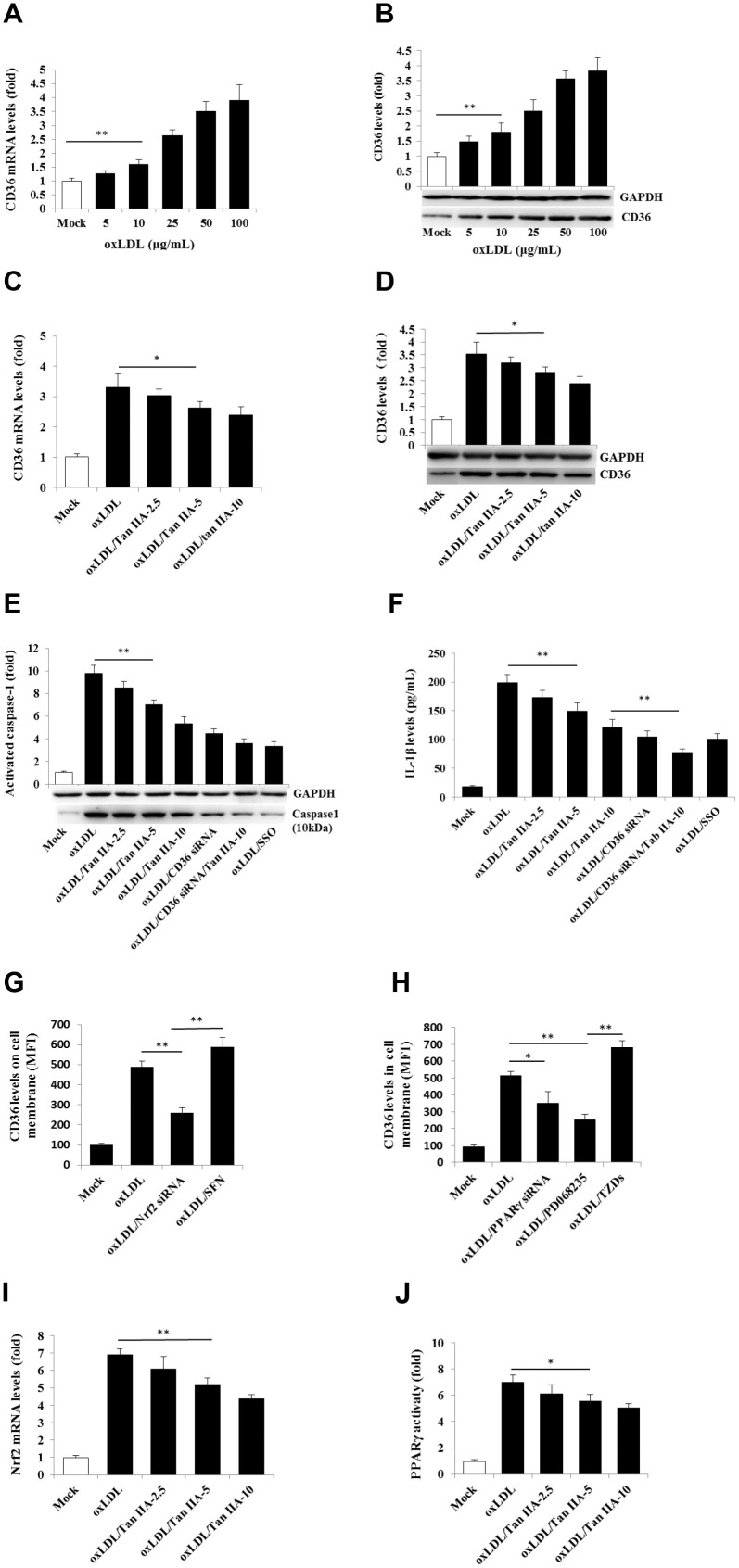
**Tan IIA inhibits oxLDL-induced CD36 expression by inhibiting Nrf2 expression and PPARγ activity.** (**A**, **B**) Effects of oxLDL on CD36 expressions at mRNA levels measured by qRT-PCR and protein levels detected by Western blot in macrophage, respectively; GAPDH, glyceraldehyde-3-phosphate dehydrogenase as an internal control. (**C**, **D**) Effects of Tan IIA on oxLDL-induced CD36 expressions at mRNA levels and protein levels in macrophages, respectively. (**E**) Effects of Tan IIA on oxLDL-induced caspase-1 activation in the cells detected by Western blot. (**F**) Effects of Tan IIA on oxLDL-induced IL-1β productions measured by ELISA in macrophages. (**G**, **H**) Effects of Nrf2 and PPARγ gene knockdown by siRNA on CD36 expression on the cell membrane, respectively, detected by flow cytometry, presented by mean fluorescence intensity (MFI). (**I**, **J**) Effects of Tan IIA on oxLDL-mediated Nrf2 expression measured by RT-PCR and oxLDL-mediated PPARγ activity measured by a commercial kit in the cells.

A number of previous studies have shown that CD36 is not only involved in oxLDL-uptaking, but also involved in intracellular cholesterol crystal formation in macrophages, leading to lysosome rupture and cytosolic release of its contents (namely cathepsins) that indirectly activate NLRP3 [41]. We therefore investigated whether Tan IIA may affect CD36 function, thereby regulating the NLRP3 inflammasome activation. We first confirmed previous observations [42, 43] that oxLDL indeed upregulated CD36 expression in macrophages (Figure 5A, 5B). Importantly, Tan IIA treatment prior to oxLDL stimulation significantly inhibited CD36 upregulation (Figure 5C, 5D). Furthermore, by using CD36 siRNAs as well as a CD36-specific inhibitor—sulfosuccinimidyl oleate (SSO), we found that inhibition of CD36 attenuated oxLDL-induced caspase 1 activation and IL-1β production (Figure 5E, 5F). These results collectively suggest that Tan IIA reduces oxLDL-induced CD36 expression in macrophages, and thereby limits subsequent NLRP3 inflammasome activation.

Next, we investigated the molecular mechanism by which Tan IIA attenuated oxLDL-induced CD36 expression. Two transcriptional factors—nuclear factor-E2-related factor 2 (Nrf2) and peroxisome proliferator-activated receptor-γ (PPARγ)—were shown to be responsible for inducing CD36 expression [44–47], we therefore tested whether inhibition of Nrf2 and PPARγ could reduce CD36 expression after oxLDL stimulation. Flow cytometry analyses showed that silencing *Nrf2* gene by siRNA significantly inhibited oxLDL-induced CD36 expression, whereas Nrf2 activator sulforaphane (SFN) enhanced CD36 expression (Figure 5G). Similarly, inhibition of PPARγ by siRNA or its pharmacological inhibitor PD 068235 significantly inhibited oxLDL-induced CD36 expression, whereas PPARγ-agonist thiazolidinediones (TZDs) promoted oxLDL-induced CD36 expression (Figure 5H). These results suggest that oxLDL-induced CD36 expression is Nrf2- and PPARγ-dependent. Of note, we found that Tan IIA could dose-dependently inhibit oxLDL-induced Nrf2 expression and PPARγ activity (Figure 5I, 5J), thereby suppressing CD36 expression and subsequent NLRP3 inflammasome activation.

### Tan IIA inhibits oxLDL-induced CD36-dependent lysosomal damage

**Figure 6:**
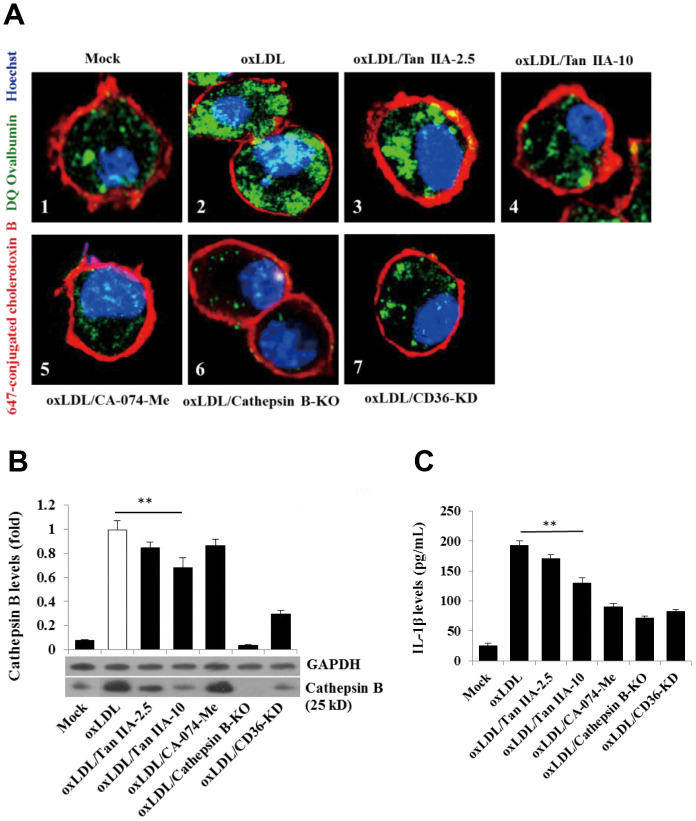
**Tan IIA inhibits oxLDL-induced macrophage lysosomal damage and cathepsin B release.** (**A**) Fluorescence confocal assay for oxLDL-induced cathepsin B release from lysosome of mouse B6 macrophages or cathepsin B^-/-^ B6 macrophages under the different treatments, respectively. The cells were treated with oxLDL (50 μg/mL) alone (2), oxLDL plus 2.5 μg/mL Tan IIA (3), oxLDL plus 10 μg/mL Tan IIA (4) and oxLDL plus Cathepsin B inhibitor CA-074-Me (5). The Cathepsin B^-/-^ mouse macrophages (6) were treated with oxLDL alone. The small interfering RNA-mediated CD36 silenced-mouse B6 macrophages (7) were treated with oxLDL alone. All the cells were stained with Alexa 647-conjugated cholera toxin B (membrane staining, red), and then with DQ ovalbumin (cathepsin B staining, green) and Hoechst dye (nuclei staining, blue) after saponin treatment. (**B**) Western blot detection for Cathepsin B releases from cytosol of oxLDL-induced B6 macrophages or cathepsin B^-/-^ B6 macrophages treated with digitonin and phenylmethylsulfonyl fluoride (PMSF). (**C**) ELISA assay for oxLDL-induced Il-1β releases from mouse B6 macrophages or cathepsin B^-/-^ B6 macrophages under the different treatments.

Previous work suggests that lysosomal damage and cathepsin B release are key events during oxLDL-induced NLRP3 inflammasome activation [15, 16]. We therefore tested whether Tan IIA directly or indirectly affected these processes to inhibit NLRP3 inflammasome activation. WT macrophages were pretreated with Tan IIA followed by stimulation with oxLDL. The cells were then stained with fluorescently labeled chlorotoxin B (red) for cell membrane, DQ Ovalbumin (green) (fluorogenic substrate for cathepsin B) for cathepsin B activity and Hoechst 33342 (blue) for nuclear after saponin treatment. Confocal imaging analysis showed that cathepsin B activity (as shown by the green fluorescent signal) in untreated WT cells (Mock) was largely restricted to cellular compartments (lysosome) (Figure 6A1). Notably, upon stimulation with oxLDL, cathepsin B started to become diffused in the cytosol (Figure 6A2), suggesting the possible rupture of lysosome that caused its release. However, when the cells were pretreated by Tan IIA, cytosolic diffuse of cathepsin B was significantly decreased (Figure 6A3, 6A4). As controls for the specificity of the above assay, we observed little green fluorescent signal in cathepsin B^-/-^ macrophages with or without ox-LDL stimulation (Figure 6A6). Additionally, CA-074-Me—the cathepsin B inhibitor—strongly decreased the green signal in cytosol of oxLDL-stimulated WT macrophages (Figure 6A5).

We also observed that CD36 is required for oxLDL-induced lysosomal damage because silencing CD36 by siRNA significantly inhibited cathepsin B cytosolic release (Figure 6A7). To further verify that Tan IIA inhibits oxLDL-induced lysosomal damage and cathepsin B release, WT macrophages were pretreated with Tan IIA and then incubated with digitonin plus phenylmethylsulfonyl fluoride (PMSF) to permeabilize the plasma membrane without disturbing the membrane integrity of cellular organelles (e.g. lysosome, ER and mitochondria). During this procedure, cathepsin B that escaped from damaged lysosome can be further released into culture medium. Immunoblot analysis showed that cathepsin B (25 kDa) was detected in oxLDL-stimulated cells, but not untreated cells (Mock) (Figure 6B). CD36 siRNA significantly inhibit cathepsin B release from lysosome in oxLDL-stimulated macrophages. Tan IIA pretreatment significantly inhibited cathepsin B release from lysosome and reduced IL-1β levels in the culture medium (Figure 6C). Together, the above results indicate that Tan IIA suppresses oxLDL-induced CD36-dependent lysosomal damage and cathepsin B release.

Since our results show that Tan IIA prevents lysosomal and mitochondrial damage after oxLDL stimulation in macrophage, we reasoned that it may possess the ability to stabilize the lipid membrane. To test this hypothesis, we performed anti-hypotonic solution-induced haemolysis assay using rat erythrocytes and anti-lipid peroxidation assay with the thiobarbital acid method by measuring malondialdehyde (MDA). As shown in Supplementary Figure 3, Tan IIA partially inhibited hypotonic solution-induced haemolysis in a dose-dependent manner, suggesting that Tan IIA is capable of stabilizing the membrane. The stability of cellular membranes largely relies on the phospholipids present in bilayer membranes. Upon oxidative stress, these phospholipids can be oxidized by ROS, resulting in lipid peroxidation that alters membrane integrity. To test if oxLDL induce lipoperoxidation and whether Tan IIA inhibits this process *in vitro*, we pretreated WT macrophages with Tan IIA for 12 h and then stimulated the cells with oxLDL for 24 h or H_2_O_2_ (as a positive control) for 2 h. We found that Tan IIA indeed significantly inhibited oxLDL- and H_2_O_2_-induced MDA increase (Supplementary Figure 4), thus indicating that Tan IIA possesses the capacity to inhibit lipid peroxidation induced by ROS.

## DISCUSSION

*Salvia miltiorrhiza*, a traditional Chinese medicine containing active ingredients of Tan IIA, is an anti-atherosclerosis drug that has been widely used in China and Southeast Asian countries for many centuries. Although some reports showed that Tan IIA can inhibit ROS-induced oxidative stress [48–51] and attenuate vascular inflammation [38], the precise molecular mechanism by which Tan IIA inhibits atherogenesis remains elusive. Our results presented here show, for the first time, that Tan IIA is a potent inhibitor of the NLRP3 inflammasome—a key pathogenic driver of atherosclerosis.

A number of recent studies have shown that sterile inflammation caused by various pathogenic factors is a key pathogenic promoter of atherosclerosis [9, 17]. Specifically, dysregulated NLRP3 inflammasome—an innate immune sensor for tissue damage—is responsible for initiation of sterile inflammation in the artery that leads to atherosclerosis [15, 16, 32, 8]. Mechanistically, the oxLDL and ChC were identified to be the pivotal activators of NLRP3 inflammasome in the context of atherosclerosis. Based on these previous findings, we speculated that Tan IIA may inhibit the NLRP3 inflammasome activation and thereby prevents the development of atherosclerosis [38–40]. In this study, we first confirmed the potent anti-atherogenic efficacy of Tan IIA using ApoE^-/-^ mice—a well-established murine atherosclerosis model [52] and then further investigated whether and how Tan IIA inhibits oxLDL-induced NLRP3 inflammasome activation in macrophages. Our results show that Tan IIA inhibits both the priming and activation steps for NLRP3 inflammasome activation. Tan IIA inhibits NF-κB activation, and thus attenuates the inflammasome priming strength. However, whether Tan IIA directly inhibits NF-κB or through some unknown indirect mechanisms remains an open question. Tan IIA also attenuates the inflammasome activation by reducing LOX-1 and CD36 expression, thereby limiting oxLDL-induced mitochondrial and lysosomal damage to ultimately inhibit NLRP3 inflammasome activation.

LOX-1 is a key scavenger receptor responsible for cellular uptake of oxLDL. Stimulation of macrophages with oxLDL induces mitochondrial stress characterized by overproduction of mtROS that oxidatively damage mitochondrial contents, including mitochondrial DNA (mtDNA) [32, 33, 53]. Oxidized mtDNA has been shown to promote atherogenesis through regulating the NLRP3 inflammasome activation [54]. Our current results demonstrated that oxLDL could induce LOX-1 expression, promote mitochondrial damage and mtROS generation and activate NLRP3 inflammasome in macrophages. Of note, we show that Tan IIA can inhibit oxLDL-induced NLRP3 activation by suppressing LOX-1 expression, although the molecular mechanism involved needs further investigation.

Tan IIA’s inhibitory effect on NLRP3 inflammasome activation also involves CD36—another scavenge receptor for cellular uptake of oxLDL in macrophages and other vascular cells [43]. It was shown that CD36 promotes oxLDL-induced NLRP3 inflammasome activation by facilitating intracellular nucleation of oxLDL-ChC into ChC particulates [15, 41], which in turn cause endo-lysosomal rupture, resulting in the cytosolic release of cathepsins that promote NLRP3 inflammasome activation [15, 41]. Our results showed that Tan IIA can reduce CD36 expression via inhibiting Nrf2 expression and PPARγ activity, thereby attenuating the NLRP3 inflammasome activation. Although our results suggest that Tan IIA may help prevent lipid peroxidation and thereby stabilizing cellular membranes (e.g. mitochondrial and lysosomal membranes), the precise molecular mechanism involved needs to be further defined.

Taken together, our work revealed that the potent anti-atherogenic effect of Tan IIA at least partially relied on its strong inhibition of oxLDL-induced NLRP3 inflammasome activation. Tan IIA significantly attenuated both the priming and activation steps during NLRP3 inflammasome assembly, and thereby decreases oxLDL-induced sterile inflammation and ultimately prevents the development of atherosclerosis.

## MATERIALS AND METHODS

### Mice and cells

Apolipoprotein E-deficient (ApoE^-/-^) male mice (C57BL/6 background), 7-week-old, were purchased from the Animal Center of Beijing University (Beijing, China). Mouse B6 macrophages and their derived gene-knockout macrophages, *Nlrp3*^−/−^, *Asc*^−/−^, *Capase-1*^−/−^, *Nlrc4*^−/−^, *Cathepsin* B^−/−^ macrophages were kind gifts from Dr. Katherine Fitzgerald (University of Massachusetts Medical School) [55].

### Reagents

Diphenyleneiodonium (DPI), 2′,7′-dichlorofluorescin diacetate (DCFH-DA), polysaccharide (LPS), ATP, CA-074-Me, Oil Red O, 2-thiobarbituric acid, sulfosuccinimidyl oleate (SSO), sulforaphane (SFN), PD068235, thiazolidinediones (TZDs) were purchased from Sigma (Sigma-Aldrich, St. Louis, MO, USA). Human LDL were from Biomedical Technologies (Stoughton, MA, USA). Ultrapure LPS and poly (dA:dT), Lipofectamine 2000, and MitoSOX^MT^ Red mitochondrial superoxide indicator were purchased from Invitrogen (Carlsbad, CA, USA). Alexa 647-conjugated cholera toxin B (red), Alexa 555-conjugated cholera toxin B (green) and DQ ovalbumin were purchased from Life Technologies (Carlsbad, CA, USA). Tanshinone IIA, CA-074-Me (cathepsin B inhibitor) and the antibodies used for immunoblotting, including rabbit anti-mouse caspase-1 p10 (M-20), rabbit anti-mouse cathepsin B (FL-339) / goat anti-rabbit IgG-AP, Armenian hamster anti-mouse IL-1β (B122) / goat anti-Armenian hamster IgG-AP, goat anti-mouse or human GAPDH (L-18) / rabbit anti-goat IgG-AP, goat anti-mouse LOX-1 polyclonal antibody, goat anti-mouse P62 polyclonal antibody, goat anti-mouse CD36 polyclonal antibody (N-15), goat anti-mouse NLRP3 antibody (M-12), donkey anti-goat IgG-HRP, donkey anti-goat IgG-FITC were purchased from Santa Cruz Biotechnology (Santa Cruz, CA, USA). The antibodies for mouse IL-1β measurement with ELISA, including anti-mouse IL-1β (capture antibody) and biotinylated rabbit anti-mouse IL-1β (detection antibody), and Avidin-HRP and the standard recombinant mouse IL-1β, were purchased from eBioscience (San Diego, CA, USA). Mouse TNFα, IL-6 and IL-18 ELISA kits were purchased from R&D Systems (Minneapolis, MN, USA). Nuclear Extraction Kit (ab113474) were from Abcam Biotechnology (Cambridge, United Kingdom). InstantOne^TM^ ELISA kit for NF-kB phosphorylated P65 assay (eBioscience). Lentivirus vectors (pLKO.1, pLKO.1-SCR, pVSV-G and pLV-CMVΔ8.9 plasmids) for delivery of siRNA were from Sigma (St. Louis, MO, USA). MitoTracker Deep Red and MitoTracker Green and Tetramethylrhodamine, methyl ester (TMRM) were from Life Technologies (Carlsbad, CA, USA).

### Mouse experiments

ApoE^-/-^ mice, housed under the specific antigen-free conditions on a 12-h light/dark cycle in the Experimental Animal Center of College of Veterinary Medicine in Hebei Agricultural University, were divided into three groups (n = 8 in each group), high cholesterol diet (HCh) group, high cholesterol diet plus Tanshinone IIA groups (HCh-Tan), and normal diet control group. The HCh and HCh-Tan groups were fed on a high cholesterol diet (21 % fat, 1.25 % cholesterol and 2 % cholic acid) for 16 weeks to induce AS, the control group were fed on a normal diet. The HCh-Tan groups were simultaneously treated with Tanshinone IIA (20mg/kg/day). Mice received the food and water *ad libitum*. All experiments in this study were approved by the Animal Ethics Committee of Hebei Agricultural University.

### Histological assessment of atherosclerosis

After 16-week feeding experiment, the mice were euthanized by intraperitoneal injection of avertin anesthetic (240 mg/kg) and infused successively with PBS to remove the blood from blood vessel and then with 4 % phosphate-buffered formaldehyde (PBF) containing 5% sucrose and 20 mM EDTA for 1 h to fix the artery (infused in left ventricular and released from a small hole cut in right atrium). After washing with PBS to remove the 4% PBF fixing solution, the entire aorta from brachiocephalic artery to iliac artery bifurcation was carefully excised. After removing the peripheral fat and adventitial tissue under a dissecting microscope, aortas were longitudinally opened with micro-surgical scissors and laid on a plate with dark background for enface presentation. After rinsing with 60 % isopropanol twice, the aorta was stained with filtered 0.3% Oil Red O in isopropanol for 20 min, and then distained with 60 % isopropanol. Finally, the images of the aortas were captured with a digital color camera. The percentage of the lesion area stained by Oil Red O to total luminal surface area was quantified using computer-assisted morphometry (NIH ImageJ software).

For histomorphometric analysis of aortic atherosclerosis, the aortas were isolated from euthanized mice by surgical operation after infused with PBS. After removing the peripheral fat and adventitial tissue under a dissecting microscope, the aortas were cross-cut into 3-4 mm fragments and the fragments were subsequently embedded vertically at different position on the tray with optical cutting temperature (OCT) compound at -20° C in a freezing microtome. The embedded aortic fragments were cut into 10 μm-thick sections which were then mounted on a glass slide. The sections on the slide were subjected to be stained successively with 0.3 % Oil Red O and Hematoxylin solution and then sealed by cover glass using aqueous mounting solution. The images of the aortic atherosclerosis were captured by a digital microscope as quantified using computer-assisted morphometry (NIH ImageJ software).

### Cell culture and stimulation

Mouse B6 macrophages were cultured in DMEM medium, supplemented with 10 % fetal bovine serum (FBS) and 100 units/mL penicillin, 100 μg/mL streptomycin, 2 mM L-glutamine at 37° C and 5 % CO_2_ as previously described [56].

For inflammasome activation assay, the B6 cells cultured in X-VIVO-15 serum-free medium (LONZA) was primed with LPS (0.25 μg/mL) for 12 h. The various regulators, including oxLDL, ATP or Tan IIA as described in individual experiments were added. The supernatants were collected for measurement of IL-1β by enzyme-linked immunosorbent assay (ELISA). The cells were collected either for cell imaging capture using confocal microscope after staining, or for immunoblot after cell lysis. For the experiments of Tan IIA anti-inflammasome activation induced by oxLDL, the B6 cells were pretreated with Tan IIA at different levels (2.5, 5.0, 10 μg/mL), and then primed by LPS and stimulated with oxLDL.

### Isolation of cytoplasmic and nuclear protein fractions

Cytoplasmic and nuclear protein fractions were isolated from mouse macrophages using a nuclear extraction kit (Abcam Biotechnology) following the manufacturer’s instructions.

### Detection of phosphorylated P65 in the cells and nucleus

Phosphorylated P65 levels in the cells, cytoplasm and nucleus were measured using InstantOne^TM^ ELISA kit for NF-kB phosphorylated P65 assay kit (eBioscience) according to the manual’s instructions.

### Cytokine assay

Mouse cytokines, including IL-1β, TNFα, IL-6 and IL-18 were measured by enzyme-linked immunosorbent assay (ELISA). The measurements of all the above cytokines, except for IL-1β, were performed using commercially available ELISA kits (R&D Systems) according to the manufactures’ instructions. The mouse IL-1β measurement was conducted with a sandwich ELISA as previously described [56]. Microtiter plates were coated with 100 μL/well of 3.5 μg/mL anti-mouse IL-1β antibodies and blocked with 5 % fat free milk in PBS-T at 37° C for 2 h. 100 μL of serially diluted standard recombinant mouse IL-1β (R&D System) or supernatant samples were added. After incubation at 37° C for 2 h on shaking and washing, 100 μL of 3 μg/mL biotinylated rabbit anti-mouse IL-1β antibody was added. After incubation and washing, 100 μL of 1:500 diluted Avidin-HRP was added and incubated at 37° C for 1 h. 100 μL/well of 3,3′,5,5′-Tetramethylbenzidine (TMB) substrate solution was added to each well and incubated at room temperature for 5 ~ 20 min for color development. The color reaction was terminated by adding 50 μL of 2 M H_2_SO_4_ stop solution. Absorbance value was read at 450 nm using an microplate reader (BioTek Instruments) within 30 min after reaction termination.

### Immunoblot analysis

The immunoblot analysis was performed as previously described [26]. Briefly, for cellular protein detection, the B6 macrophages in a 6-well plate were collected and lysed with 200 μL of lysis buffer (5 mM Tris-HCl, 25 mM KCl, 2 mM EGTA, 2 mM EDTA, 1 % NP-40, 150 mM NaCl, protease inhibitor cocktail in PBS, pH 7.4). The lysate supernatant was collected by centrifugation and subjected to be separated by running 15 % SDS-PAGE and transferred onto nitrocellulose membranes by electro-blotting. The protein on the membrane was detected by corresponding primary antibody and HRP- or AP-conjugated secondary antibody. For the secreted protein detection in the cell culture, the cell culture was first concentrated by trichloroacetic acid (TAC) precipitation and then subjected to immunoblot as described above.

### qRT-PCR

Total RNA was isolated from the mouse B6 macrophages with the different treated conditions using Trizol reagent (Invitrogen). The cDNA was prepared using the iScript cDNA Synthesis Kit (Bio-Rad). The mRNA levels for the different proteins were measured by qRT-PCR using the different specific primers (Supplementary Table 1). The qRT-PCR was performed using iQ SYBR Green Super-mix (Bio-Rad) with the iCycler iQ RT-PCR detection system (Bio-Rad), with 40 cycles of real-time data collection at 95° C for 30s, 50° C- 65° C (variable based on the different genes) for 1 min and 72° C for 1 min, followed by melt-curve analysis to verify the presence of a single product. All gene expression data are presented as the expression relative to GAPDH.

### RNA interference

Down-regulating LOX-1, CD36, SR-A1, Nrf2 and PPARγ expressions in mouse B6 macrophages was achieved by adopting small interfering RNA strategy using lentivirus vector as a gene delivering system. Briefly, the target sequences for each gene were selected based on their cDNA sequences using siDirect version 2.0 software (Supplementary Table 2). The double-stranded sticky-ended DNA fragments were designed based on the target sequences in order to insert the fragments into the lentivirus cloning vector (pLKO.1) at *Age* I and *Eco*R I sites to express the specific single-stranded RNA consisting of two complementary siRNA sequences linked by a loop. The pairs of oligos were synthesized (Sangon, Shanghai, China) and double-stranded DNA with 5´-*Age* I and 3´-*Eco*R I sticky ends was generated by annealing the two oligos, and inserted into the pLKO.1 plasmid through *Age* I and *Eco*R I sites to produce pLKO-X-siRNA vectors (X presents LOX-1, CD36, SR-A1, Nrf2 or PPARγ). The pLKO-X-siRNA vectors (10 μg), as well as pLKO.1-SCR vector as siRNA negative control, were co-transfected with pVSV-G (1 μg) and pLV-CMVΔ8.9 (5 μg) package vectors into HEK 293T cells cultured in OptiMEM medium (Gibco, NY, USA) in T25 flask, using Lipofectamine 2000 (20 μL) for 12 h. The medium was replaced by DMEM/15% FBS and the cells were incubated for an additional 48 h to generate recombinant lentiviruses (mouse X-siRNA-Lentiviruses and siRNA control Lentivirus), which were harvested from the culture medium, filtered with a 0.22 μm syringe filter (Millipore, Billerica, MA, USA), and titrated before use.

### ROS detection

ROS in the macrophages was detected with 2,7-dichlorofluorescein diacetate (DCFH-DA) fluorescence assay. The B6 cells in 6-well plate were washed with PBS and incubated with 10 μmol/L 2′-7′-dichlorofluorescin diacetate (DCFH-DA) in the dark at 37° C for 30 min. After carefully washing with PBS, ROS in the cells were detected using a fluorescence microscope. The mitochondria-derived ROS was measured by flow cytometry as previously described [56]. MitoSOX, a dye that stains superoxide specifically generated from mitochondria, was used to quantify the relative amounts of mitochondrial ROS after stimulations. Briefly, B6 cells were first pretreated with different doses of Tan IIA for 12 h, then stimulated with oxLDL (50 μg/mL) for 24 h, and finally loaded with 2.5 μmol/L of MitoSOX for 20 min. The cells were then washed three times with PBS and the level of mitochondrial ROS was measured via the FACSCaliber flow cytometer (BD Biosciences, San Jose, CA) and the data collected using CellQuest-Pro. Files were further analyzed using FlowJo software.

### Mitochondria damage detection

Mitochondrial damage was examined by flow cytometry using MitoTracker Deep Red and MitoTracker Green. MitoTracker Deep Red is a red fluorescent dye that stains mitochondria of living cells; MitoTracker Green is a green fluorescent dye that stains mitochondria of all cells. Mouse B6 macrophages cultured in 6-well plates were treated with different substances and then stained with 0.5 μM MitoTracker Deep Red and 0.2 μM MitoTracker Green for 30 min at 37° C in the dark. After incubation, cells were washed twice gently with PBS to remove excess unbound dye and reincubated with fresh PBS. Damage to mitochondria was measured in a FACSCaliber flow cytometer and analyzed using CellQuest software.

### Mitochondrial membrane potential (ΔΨm) loss detection

Mitochondrial membrane potential (ΔΨm) was determined using TMRM, a cell-permeant, cationic and fluorescent red-orange dye, which readily enters the cell and is then sequestered by active mitochondria (with normal Ψm). The fluorescence signal is therefore very weak. During mitochondrial damage, the ΔΨm is disturbed, the TMRM cannot be sequestered, and fluorescence can be detected. The procedure, briefly, was as follows: the B6 cells were trypsinized, resuspended in 100 μL 200 nM TMRM in a 1.5 mL tube at a cell density of 5×10^6^ cells/mL, and incubated at 37° C for 30 min before staining. After washing with washing buffer (PBS/ 50 nM TMRM), the cells were resuspended in 340 μL PBS and 100 μL aliquots of each sample in triplicate were added to wells of a 96-well plate. The fluorescent intensities were measured by spectrofluorophotometry (excitation 540 nm, emission 574 nm).

### Detection of cathepsin B release from lysosome

Two methods were used to analyze cathepsin B release from lysosome, the immunoblot for detecting the cathepsin B in the culture medium released from cytosol and the confocal imaging for intracellular cathepsin B.

Immunoblot assay was performed as previously described [24]. Briefly, the B6 cells or cathepsin B^-/-^ B6 cells were grown in 6-well plate in DMEM complete medium. When the cells reached about 85% confluence, the culture medium was changed for X-VIVO-5 serum-free medium. The cells were pretreated with Tan IIA (2.5, 10 μg/mL), CD36 siRNA-lentivirus (MOI = 30) for 12 h, or CA-074-Me (cathepsin B inhibitor, 50 μM) for 6 h, and then stimulated with oxLDL (50 μg/mL) for 24 h. The cells were collected and subjected to permeabilization with 50 μg/mL digitonin and 1 mM phenylmethylsulfonyl fluoride in PBS (pH 7.4) for 10 min on ice. This procedure has been shown to successfully lyse the plasma membrane while still keeping the integrity of membranes of intracellular organelles. The cellular extract was then subjected to immunoblot analysis for detecting cathepsin B.

For confocal imaging assay, the cells seeded on the cover slip in 24-well plate with X-VIVO-5 medium were pretreated separately with Tan IIA, CD36 siRNA or CA-074-Me, then stimulated with oxLDL as described above. The cells were fixed with 4% polyformaldehyde (PFA) at room temperature for 30 min and incubated with 1 mg/mL 647-conjugated cholera toxin B (red) at 4° C in the dark for 1h for cell membrane staining, and then treated with 0.2% saponin/PBS solution for 20 min to increase the permeability of the membrane. Finally, the cells were incubated with 0.2 mg/mL DQ Ovalbumin for cathepsin B staining (green) and 1 μg/mL Hoechst 33342 blue dye in the dark for 1h for nuclear DNA staining. After staining, the cells were imaged immediately on confocal microscope.

### Measurements of TCh, HDL-Ch, LDL-Ch and TG in mouse sera

Total Ch and triglyceride (TG) in the mouse serum were measured by end-point method with commercial kits from Beijing Lideman Biochemical (Beijing, China); High-density-lipoprotein (HDL) Ch and low-density-lipoprotein (LDL) Ch were measured by direct method with the kit from Beijing Strong Biotechnologies (Beijing, China) on the HITACHI 7600-020 chemistry analyzer (Tokyo, Japan).

### Membrane stabilizing activity assay

Membrane stabilizing activity of Tan IIA was assessed using the hypotonic solution-induced rat erythrocyte hemolysis method [57]. The rat blood was collected by cardiac puncture with heparinized syringes. The blood was washed and resuspended with the isotonic buffered solution (150 mM NaCl in 10 mM sodium phosphate buffered saline, pH 7.4). The rat erythrocyte suspension (0.5 mL) was mixed with 5 mL of hypotonic solution (50 mM NaCl in 10 mM sodium phosphate buffered saline) containing the Tan IIA (0, 2.5, 5, 10 μg/mL). The mixtures were incubated for 10 min at room temperature and centrifuged at 3000×g for 10 min and the absorbance of the supernatant was measured at 540 nm. The percentage inhibition of haemolysis or membrane stabilization was calculated according to modified method described by Shinde [57] as follows: % Inhibition of heamolysis = ((*OD*_control_ -*OD*_test_)/*OD*_control_)×100.

### Preparation of oxLDL

The oxLDL preparation was performed as previously described [41, 42]. Briefly, LDL (Biomedical Technologies) was diluted in PBS to a concentration of 250 μg /ml and was dialyzed overnight at 4° C in a 3 liters of PBS. Moderately oxidized LDL was prepared by dialysis for 6 h against 5μM CuSO_4_, which was terminated by the addition of EDTA (200 nM) and butylated hydroxytoluence (500 nM). To obtain minimally modified LDL and extensively oxidized LDL, dialysis against 5 μM CuSO_4_ was terminated after 2 h and 24 h, respectively.

### Anti-lipoperoxidation assay

Anti-lipoperoxidation of Tan IIA was detected by thiobarbituric acid (TBA) method to measure malondialdehyde (MDA) concentration using a commercial kit according to the manufacture’s instruction.

### Statistical analysis

The significance of differences between groups was evaluated by one-way analysis of variance (ANOVA) with Dunnett’s post-comparison test for multiple groups to control group, or by Student’s *t* test for two groups. All the presented data are shown as mean ±SD. *P < 0.05, **P < 0.01. NS, no significance.

## Supplementary Material

Supplementary Figures

Supplementary Tables

## References

[1] Shang Q, Xu H, Huang L. Tanshinone IIA: a promising natural cardioprotective agent. Evid Based Complement Alternat Med. 2012; 2012:716459. 10.1155/2012/716459 22454677 PMC3292221

[2] Gao S, Liu Z, Li H, Little PJ, Liu P, Xu S. Cardiovascular actions and therapeutic potential of tanshinone IIA. Atherosclerosis. 2012; 220:3–10. 10.1016/j.atherosclerosis.2011.06.041 21774934

[3] Chen Z, Xu H. Anti-inflammatory and immunomodulatory mechanism of tanshinone IIA for atherosclerosis. Evid Based Complement Alternat Med. 2014; 2014:267976. 10.1155/2014/267976 25525444 PMC4267215

[4] Weber C, Noels H. Atherosclerosis: current pathogenesis and therapeutic options. Nat Med. 2011; 17:1410–22. 10.1038/nm.2538 22064431

[5] Chen GY, Nuñez G. Sterile inflammation: sensing and reacting to damage. Nat Rev Immunol. 2010; 10:826–37. 10.1038/nri2873 21088683 PMC3114424

[6] Abbate A, Toldo S, Marchetti C, Kron J, Van Tassell BW, Dinarello CA. Interleukin-1 and the inflammasome as therapeutic targets in cardiovascular disease. Circ Res. 2020; 126:1260–80. 10.1161/CIRCRESAHA.120.315937 32324502 PMC8760628

[7] Hu Q, Wei B, Wei L, Hua K, Yu X, Li H, Ji H. Sodium tanshinone IIA sulfonate ameliorates ischemia-induced myocardial inflammation and lipid accumulation in beagle dogs through NLRP3 inflammasome. Int J Cardiol. 2015; 196:183–92. 10.1016/j.ijcard.2015.05.152 26143630

[8] Grebe A, Hoss F, Latz E. NLRP3 inflammasome and the IL-1 pathway in atherosclerosis. Circ Res. 2018; 122:1722–40. 10.1161/CIRCRESAHA.118.311362 29880500

[9] Rock KL, Latz E, Ontiveros F, Kono H. The sterile inflammatory response. Annu Rev Immunol. 2010; 28:321–42. 10.1146/annurev-immunol-030409-101311 20307211 PMC4315152

[10] Moore KJ, Tabas I. Macrophages in the pathogenesis of atherosclerosis. Cell. 2011; 145:341–55. 10.1016/j.cell.2011.04.005 21529710 PMC3111065

[11] Parsamanesh N, Moossavi M, Bahrami A, Fereidouni M, Barreto G, Sahebkar A. NLRP3 inflammasome as a treatment target in atherosclerosis: a focus on statin therapy. Int Immunopharmacol. 2019; 73:146–55. 10.1016/j.intimp.2019.05.006 31100709

[12] Patel MN, Carroll RG, Galván-Peña S, Mills EL, Olden R, Triantafilou M, Wolf AI, Bryant CE, Triantafilou K, Masters SL. Inflammasome priming in sterile inflammatory disease. Trends Mol Med. 2017; 23:165–80. 10.1016/j.molmed.2016.12.007 28109721

[13] Baldrighi M, Mallat Z, Li X. NLRP3 inflammasome pathways in atherosclerosis. Atherosclerosis. 2017; 267:127–38. 10.1016/j.atherosclerosis.2017.10.027 29126031

[14] Lukens JR, Gross JM, Kanneganti TD. IL-1 family cytokines trigger sterile inflammatory disease. Front Immunol. 2012; 3:315. 10.3389/fimmu.2012.00315 23087690 PMC3466588

[15] Duewell P, Kono H, Rayner KJ, Sirois CM, Vladimer G, Bauernfeind FG, Abela GS, Franchi L, Nuñez G, Schnurr M, Espevik T, Lien E, Fitzgerald KA, et al. NLRP3 inflammasomes are required for atherogenesis and activated by cholesterol crystals. Nature. 2010; 464:1357–61. 10.1038/nature08938 20428172 PMC2946640

[16] Rajamäki K, Lappalainen J, Oörni K, Välimäki E, Matikainen S, Kovanen PT, Eklund KK. Cholesterol crystals activate the NLRP3 inflammasome in human macrophages: a novel link between cholesterol metabolism and inflammation. PLoS One. 2010; 5:e11765. 10.1371/journal.pone.0011765 20668705 PMC2909263

[17] Cassel SL, Sutterwala FS. Sterile inflammatory responses mediated by the NLRP3 inflammasome. Eur J Immunol. 2010; 40:607–11. 10.1002/eji.200940207 20201012 PMC3601805

[18] Martinon F, Burns K, Tschopp J. The inflammasome: a molecular platform triggering activation of inflammatory caspases and processing of proIL-beta. Mol Cell. 2002; 10:417–26. 10.1016/s1097-2765(02)00599-3 12191486

[19] Latz E, Xiao TS, Stutz A. Activation and regulation of the inflammasomes. Nat Rev Immunol. 2013; 13:397–411. 10.1038/nri3452 23702978 PMC3807999

[20] Rathinam VA, Vanaja SK, Fitzgerald KA. Regulation of inflammasome signaling. Nat Immunol. 2012; 13:333–42. 10.1038/ni.2237 22430786 PMC3523703

[21] Tschopp J, Schroder K. NLRP3 inflammasome activation: the convergence of multiple signalling pathways on ROS production? Nat Rev Immunol. 2010; 10:210–15. 10.1038/nri2725 20168318

[22] Mariathasan S, Weiss DS, Newton K, McBride J, O’Rourke K, Roose-Girma M, Lee WP, Weinrauch Y, Monack DM, Dixit VM. Cryopyrin activates the inflammasome in response to toxins and ATP. Nature. 2006; 440:228–32. 10.1038/nature04515 16407890

[23] Gurcel L, Abrami L, Girardin S, Tschopp J, van der Goot FG. Caspase-1 activation of lipid metabolic pathways in response to bacterial pore-forming toxins promotes cell survival. Cell. 2006; 126:1135–45. 10.1016/j.cell.2006.07.033 16990137

[24] Li W, Chang Y, Liang S, Zhong Z, Li X, Wen J, Zhang Y, Zhang J, Wang L, Lin H, Cao X, Huang H, Zhong F. NLRP3 inflammasome activation contributes to listeria monocytogenes-induced animal pregnancy failure. BMC Vet Res. 2016; 12:36. 10.1186/s12917-016-0655-2 26911557 PMC4765044

[25] Ichinohe T, Pang IK, Iwasaki A. Influenza virus activates inflammasomes via its intracellular M2 ion channel. Nat Immunol. 2010; 11:404–10. 10.1038/ni.1861 20383149 PMC2857582

[26] Zhang K, Hou Q, Zhong Z, Li X, Chen H, Li W, Wen J, Wang L, Liu W, Zhong F. Porcine reproductive and respiratory syndrome virus activates inflammasomes of porcine alveolar macrophages via its small envelope protein E. Virology. 2013; 442:156–62. 10.1016/j.virol.2013.04.007 23664331

[27] Martinon F, Pétrilli V, Mayor A, Tardivel A, Tschopp J. Gout-associated uric acid crystals activate the NALP3 inflammasome. Nature. 2006; 440:237–41. 10.1038/nature04516 16407889

[28] Chu J, Thomas LM, Watkins SC, Franchi L, Núñez G, Salter RD. Cholesterol-dependent cytolysins induce rapid release of mature IL-1beta from murine macrophages in a NLRP3 inflammasome and cathepsin b-dependent manner. J Leukoc Biol. 2009; 86:1227–38. 10.1189/jlb.0309164 19675207 PMC2774880

[29] Zhao L, Varghese Z, Moorhead JF, Chen Y, Ruan XZ. CD36 and lipid metabolism in the evolution of atherosclerosis. Br Med Bull. 2018; 126:101–12. 10.1093/bmb/ldy006 29534172

[30] Crucet M, Wüst SJ, Spielmann P, Lüscher TF, Wenger RH, Matter CM. Hypoxia enhances lipid uptake in macrophages: role of the scavenger receptors Lox1, SRA, and CD36. Atherosclerosis. 2013; 229:110–17. 10.1016/j.atherosclerosis.2013.04.034 23706521

[31] Singh S, Gautam AS. Upregulated LOX-1 receptor: key player of the pathogenesis of atherosclerosis. Curr Atheroscler Rep. 2019; 21:38. 10.1007/s11883-019-0801-y 31350594

[32] Ding Z, Liu S, Wang X, Dai Y, Khaidakov M, Deng X, Fan Y, Xiang D, Mehta JL. LOX-1, mtDNA damage, and NLRP3 inflammasome activation in macrophages: implications in atherogenesis. Cardiovasc Res. 2014; 103:619–28. 10.1093/cvr/cvu114 24776598 PMC4200051

[33] Christ A, Latz E. LOX-1 and mitochondria: an inflammatory relationship. Cardiovasc Res. 2014; 103:435–37. 10.1093/cvr/cvu187 25114006

[34] Zhong Z, Umemura A, Sanchez-Lopez E, Liang S, Shalapour S, Wong J, He F, Boassa D, Perkins G, Ali SR, McGeough MD, Ellisman MH, Seki E, et al. NF-κB restricts inflammasome activation via elimination of damaged mitochondria. Cell. 2016; 164:896–910. 10.1016/j.cell.2015.12.057 26919428 PMC4769378

[35] Zhong Z, Liang S, Sanchez-Lopez E, He F, Shalapour S, Lin XJ, Wong J, Ding S, Seki E, Schnabl B, Hevener AL, Greenberg HB, Kisseleva T, Karin M. New mitochondrial DNA synthesis enables NLRP3 inflammasome activation. Nature. 2018; 560:198–203. 10.1038/s41586-018-0372-z 30046112 PMC6329306

[36] Zhou R, Yazdi AS, Menu P, Tschopp J. A role for mitochondria in NLRP3 inflammasome activation. Nature. 2011; 469:221–25. 10.1038/nature09663 21124315

[37] Terada K, Yamada J, Hayashi Y, Wu Z, Uchiyama Y, Peters C, Nakanishi H. Involvement of cathepsin B in the processing and secretion of interleukin-1beta in chromogranin A-stimulated microglia. Glia. 2010; 58:114–24. 10.1002/glia.20906 19544382

[38] Jang SI, Kim HJ, Kim YJ, Jeong SI, You YO. Tanshinone IIA inhibits LPS-induced NF-kappaB activation in RAW 264.7 cells: possible involvement of the NIK-IKK, ERK1/2, p38 and JNK pathways. Eur J Pharmacol. 2006; 542:1–7. 10.1016/j.ejphar.2006.04.044 16797002

[39] Wang P, Wu X, Bao Y, Fang J, Zhou S, Gao J, Pi R, Mou YG, Liu P. Tanshinone IIA prevents cardiac remodeling through attenuating NAD (P)H oxidase-derived reactive oxygen species production in hypertensive rats. Pharmazie. 2011; 66:517–24. 21812327

[40] Tang FT, Cao Y, Wang TQ, Wang LJ, Guo J, Zhou XS, Xu SW, Liu WH, Liu PQ, Huang HQ. Tanshinone IIA attenuates atherosclerosis in ApoE(-/-) mice through down-regulation of scavenger receptor expression. Eur J Pharmacol. 2011; 650:275–84. 10.1016/j.ejphar.2010.07.038 20854809

[41] Sheedy FJ, Grebe A, Rayner KJ, Kalantari P, Ramkhelawon B, Carpenter SB, Becker CE, Ediriweera HN, Mullick AE, Golenbock DT, Stuart LM, Latz E, Fitzgerald KA, Moore KJ. CD36 coordinates NLRP3 inflammasome activation by facilitating intracellular nucleation of soluble ligands into particulate ligands in sterile inflammation. Nat Immunol. 2013; 14:812–20. 10.1038/ni.2639 23812099 PMC3720827

[42] D’Archivio M, Scazzocchio B, Filesi C, Varì R, Maggiorella MT, Sernicola L, Santangelo C, Giovannini C, Masella R. Oxidised LDL up-regulate CD36 expression by the Nrf2 pathway in 3T3-L1 preadipocytes. FEBS Lett. 2008; 582:2291–98. 10.1016/j.febslet.2008.05.029 18514070

[43] Ishii T, Itoh K, Ruiz E, Leake DS, Unoki H, Yamamoto M, Mann GE. Role of Nrf2 in the regulation of CD36 and stress protein expression in murine macrophages: activation by oxidatively modified LDL and 4-hydroxynonenal. Circ Res. 2004; 94:609–16. 10.1161/01.RES.0000119171.44657.45 14752028

[44] Freigang S, Ampenberger F, Spohn G, Heer S, Shamshiev AT, Kisielow J, Hersberger M, Yamamoto M, Bachmann MF, Kopf M. Nrf2 is essential for cholesterol crystal-induced inflammasome activation and exacerbation of atherosclerosis. Eur J Immunol. 2011; 41:2040–51. 10.1002/eji.201041316 21484785

[45] Zhao C, Gillette DD, Li X, Zhang Z, Wen H. Nuclear factor E2-related factor-2 (Nrf2) is required for NLRP3 and AIM2 inflammasome activation. J Biol Chem. 2014; 289:17020–29. 10.1074/jbc.M114.563114 24798340 PMC4059144

[46] Olagnier D, Lavergne RA, Meunier E, Lefèvre L, Dardenne C, Aubouy A, Benoit-Vical F, Ryffel B, Coste A, Berry A, Pipy B. Nrf2, a PPARγ alternative pathway to promote CD36 expression on inflammatory macrophages: implication for malaria. PLoS Pathog. 2011; 7:e1002254. 10.1371/journal.ppat.1002254 21949655 PMC3174257

[47] Ren J, Jin W, Chen H. oxHDL decreases the expression of CD36 on human macrophages through PPARgamma and p38 MAP kinase dependent mechanisms. Mol Cell Biochem. 2010; 342:171–81. 10.1007/s11010-010-0481-y 20458524

[48] Zhang HS, Wang SQ. Nrf2 is involved in the effect of tanshinone IIA on intracellular redox status in human aortic smooth muscle cells. Biochem Pharmacol. 2007; 73:1358–66. 10.1016/j.bcp.2007.01.004 17303087

[49] Chen W, Tang F, Xie B, Chen S, Huang H, Liu P. Amelioration of atherosclerosis by tanshinone IIA in hyperlipidemic rabbits through attenuation of oxidative stress. Eur J Pharmacol. 2012; 674:359–64. 10.1016/j.ejphar.2011.10.040 22088276

[50] Tang F, Wu X, Wang T, Wang P, Li R, Zhang H, Gao J, Chen S, Bao L, Huang H, Liu P. Tanshinone II a attenuates atherosclerotic calcification in rat model by inhibition of oxidative stress. Vascul Pharmacol. 2007; 46:427–38. 10.1016/j.vph.2007.01.001 17337361

[51] Li YI, Elmer G, Leboeuf RC. Tanshinone IIA reduces macrophage death induced by hydrogen peroxide by upregulating glutathione peroxidase. Life Sci. 2008; 83:557–62. 10.1016/j.lfs.2008.08.003 18762198 PMC2613784

[52] Zhang SH, Reddick RL, Piedrahita JA, Maeda N. Spontaneous hypercholesterolemia and arterial lesions in mice lacking apolipoprotein E. Science. 1992; 258:468–71. 10.1126/science.1411543 1411543

[53] Gao S, Geng YJ. LOX-1: a male hormone-regulated scavenger receptor for atherosclerosis. Vascul Pharmacol. 2013; 59:138–43. 10.1016/j.vph.2013.10.003 24157503

[54] Tumurkhuu G, Shimada K, Dagvadorj J, Crother TR, Zhang W, Luthringer D, Gottlieb RA, Chen S, Arditi M. Ogg1-dependent DNA repair regulates NLRP3 inflammasome and prevents atherosclerosis. Circ Res. 2016; 119:e76–90. 10.1161/CIRCRESAHA.116.308362 27384322 PMC5010464

[55] Hornung V, Bauernfeind F, Halle A, Samstad EO, Kono H, Rock KL, Fitzgerald KA, Latz E. Silica crystals and aluminum salts activate the NALP3 inflammasome through phagosomal destabilization. Nat Immunol. 2008; 9:847–56. 10.1038/ni.1631 18604214 PMC2834784

[56] Zhong Z, Zhai Y, Liang S, Mori Y, Han R, Sutterwala FS, Qiao L. TRPM2 links oxidative stress to NLRP3 inflammasome activation. Nat Commun. 2013; 4:1611. 10.1038/ncomms2608 23511475 PMC3605705

[57] Shinde UA, Phadke AS, Nair AM, Mungantiwar AA, Dikshit VJ, Saraf MN. Membrane stabilizing activity-a possible mechanism of action for the anti-inflammatory activity of Cedrus deodara wood oil. Fitoterapia. 1999; 70:251–257. 10.1016/S0367-326X(99)00030-1

