# Supplementary material for: Tanshinone IIA attenuates atherosclerosis via inhibiting NLRP3 inflammasome activation
Source: Aging (Albany NY). 2020 Nov 16;13(1):910–32. doi: 10.18632/aging.202202 (PMC7835056; doi:10.18632/aging.202202)
Supplement: Supplementary Figures [file aging-13-202202-s001.pdf]

SUPPLEMENTARY FIGURES

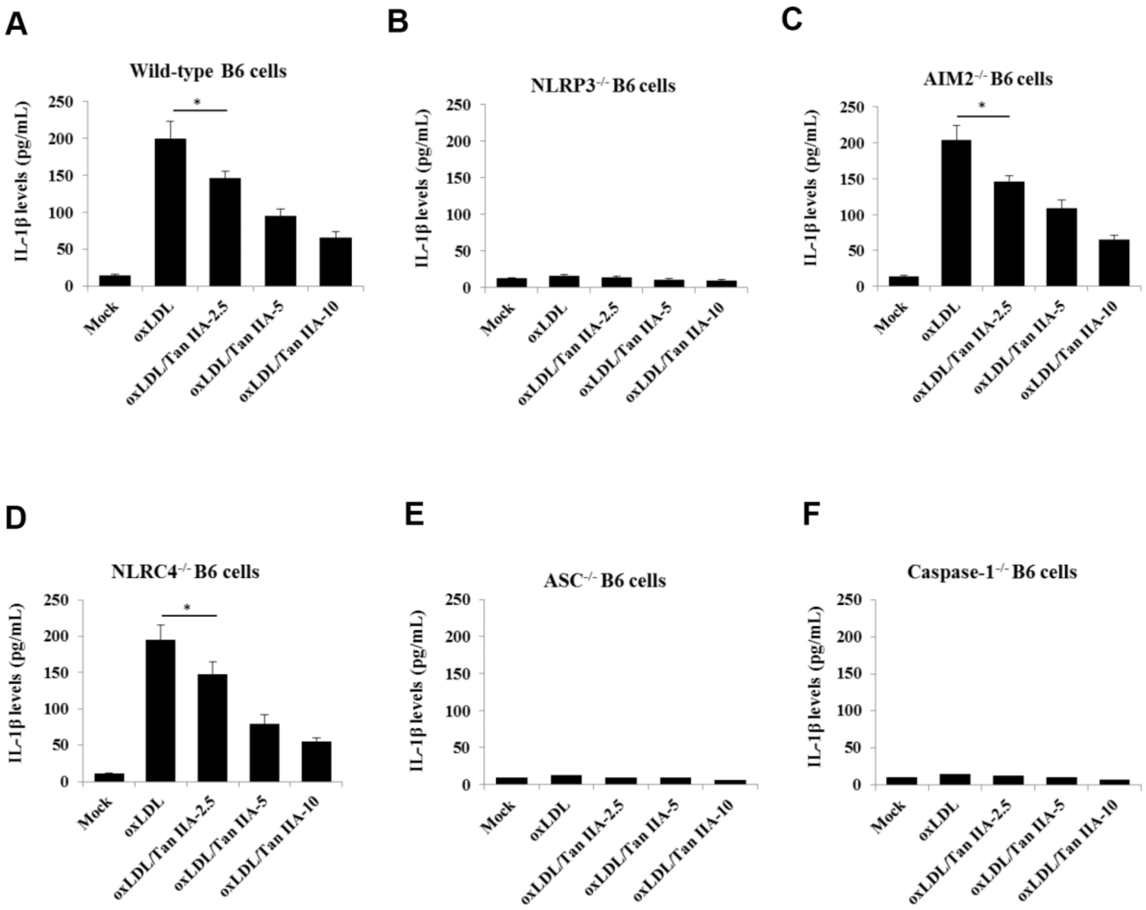

Supplementary Figure 1. Inhibitory effects of Tan IIA on IL-β release from oxLDL-induced different sensor gene-knockout macrophages. (**A**–**F**) Wild-type, NLRP3-/-, AIM-/-, NLRC4-/-, ASC-/- and caspase 1-/- mouse B6 macrophages.

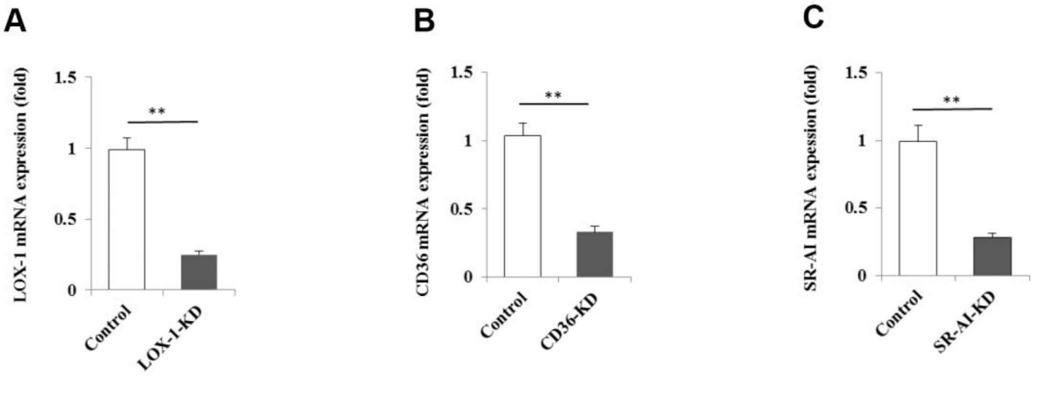

Supplementary Figure 2. Effects of the specific siRNAs on LOX-1, CD36 and SR-A1 expressions. (**A**–**C**) LOX-1, SR-A1 and CD36 expressions measured by qRT-PCR in the macrophages under the treatments with their siRNA, respectively.

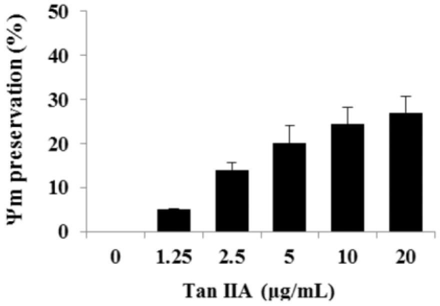

Supplementary Figure 3. Inhibition rates of Tan IIA on hypotonic solution-induced rat erythrocyte hemolysis.

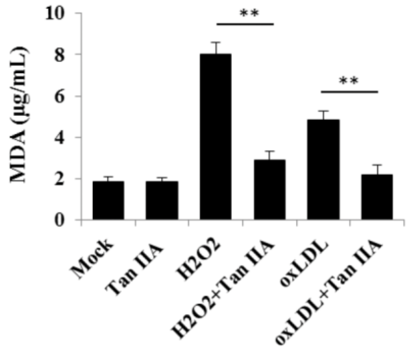

Supplementary Figure 4. Tan IIA inhibition on lipid peroxidation induced by H2O2.
