# Supplementary material for: Tanshinone IIA attenuates atherosclerosis via inhibiting NLRP3 inflammasome activation
Source: Aging (Albany NY). 2020 Nov 16;13(1):910–32. doi: 10.18632/aging.202202 (PMC7835056; doi:10.18632/aging.202202)
Supplement: Supplementary Tables [file aging-13-202202-s002.pdf]

SUPPLEMENTARY TABLES

Supplementary Table 1. qRT-PCR primers.

Genes	Primers sequences (5′→3′)		Size (bp)	Accessary No.
	Forward	Reverse		
IL-1β	AATGCCACCTTTTGACAGTGATG	GGAAGGTCCACGGGAAAGAC	224	NM_008361.4
TNFα	AGGCACTCCCCCAAAAGATG	CCACTTGGTGGTTTGTGAGTG	213	NM_001278601.1
IL-6	GCCTTCTTGGGACTGATGCT	TGGAAATTGGGGTAGGAAGGAC	475	NM_031168.2
IL-18	GGCTGCCATGTCAGAAGACT	GTCTGGTCTGGGGTTCACTG	239	NM_008360.2
NLRP3	CTACGGCCGTCTACGTCTTC	GGCCAAAGAGGAATCGGACA	438	NM_145827.4
LOX-1	GCGAACCTTACTCAGCAGGA	GGGCCCATGGAAGAGGTAAC	546	NM_138648.2
CD36	GGAGGCATTCTCATGCCAGT	CTGCTGTTCTTTGCCACGTC	176	NM_001159556.1
SR-A1	TGTGGCTCTACCCCTCCTAC	GCAGATCTTGTGGACAGCCT	231	NM_001008422.1
Nrf2	CCTCTGTCACCAGCTCAAGG	TTCTGGGCGGCGACTTTATT	323	NM_010902.4

Supplementary Table 2. Small interfering RNA target sequences and double-strand DNA for generating siRNAs.

Genes	Target sequence	Location	Double-stranded DNA designed for insertion into cloning vector*
LOX-1	GGTGTTGTCAGTGACC CTTAT	185-205	5′ CCGGGGTGTTGTCAGTGACCCTTATCTCGAGATAAGGGTCACTGACAACAC CTTTTTG CCACAACAGTCACTGGGAATAGAGCTCTATTCCCAGTGACTGTTGTGGAAAAACTTA A5′
CD36	GGAGCCATCTTTGAG CCTTCA	818-838	5′ CCGGGGAGCCATCTTTGAGCCTTCACTCGAGTGAAGGCTCAAAGATGGCTCC TTTTTG CCTCGGTAGAAACTCGGAAGTGAGCTCACTTCCGAGTTTCTACCGAGGAAAAACTTA A5′
SR-A1	GGGATAGGTCATCC AAGAAGA	2386-2406	5′ CCGGGGGATAGGTCATCCAAGAAGACTCGAGTCTTCTTGGATGACCTATCCC TTTTTG CCCTATCCAGTAGGTTCTTCTGAGCTCAGAAGAACCTACTGGATAGGGAAAAACTTA A5′
Nrf2	GCAGGACATGGATT TGATTGA	297-317	5′ CCGGGCAGGACATGGATTTGATTGACTCGAGTCAATCAAATCCATGTCCTGC TTTTTG CGTCCTGTACCTAAACTAACTGAGCTCAGTTAGTTTAGGTACAGGACGAAAAACTTA A5′
PPARγ	GCATTTCTGCTCCAC ACTATG	618-639	5′ CCGGGCATTTCTGCTCCACACTATGCTCGAGCATAGTGTGGAGCAGAAATGC TTTTTG CGTAAAGACGAGGTGTCATACGAGCTCGTATCACACCTCGTCTTTACGAAAAACTTA A5′

* Cloning sites: *Age* I/*Eco*R I.
